# The relationship between motor pathway damage and flexion-extension patterns of muscle co-excitation during walking

**DOI:** 10.3389/fneur.2022.968385

**Published:** 2022-10-28

**Authors:** Shraddha Srivastava, Bryant A. Seamon, Barbara K. Marebwa, Janina Wilmskoetter, Mark G. Bowden, Chris M. Gregory, Na Jin Seo, Colleen A. Hanlon, Leonardo Bonilha, Truman R. Brown, Richard R. Neptune, Steven A. Kautz

**Affiliations:** ^1^Ralph H. Johnson VA Medical Center, Charleston, SC, United States; ^2^Department of Health Sciences and Research, College of Health Professions, Medical University of South Carolina, Charleston, SC, United States; ^3^Division of Physical Therapy, Department of Rehabilitation Sciences, College of Health Professions, Medical University of South Carolina, Charleston, SC, United States; ^4^Department of Neurology, College of Medicine, Medical University of South Carolina, Charleston, SC, United States; ^5^Division of Occupational Therapy, College of Health Professions, Medical University of South Carolina, Charleston, SC, United States; ^6^Department of Cancer Biology, Wake Forest School of Medicine, Winston-Salem, NC, United States; ^7^Department of Radiology and Radiological Science, College of Medicine, Medical University of South Carolina, Charleston, SC, United States; ^8^Walker Department of Mechanical Engineering, The University of Texas at Austin, Austin, TX, United States

**Keywords:** stroke, gait, corticospinal tract (CST), corticoreticular pathways (CRP), EMG, muscle modules, abnormal synergies

## Abstract

**Background:**

Mass flexion-extension co-excitation patterns during walking are often seen as a consequence of stroke, but there is limited understanding of the specific contributions of different descending motor pathways toward their control. The corticospinal tract is a major descending motor pathway influencing the production of normal sequential muscle coactivation patterns for skilled movements. However, control of walking is also influenced by non-corticospinal pathways such as the corticoreticulospinal pathway that possibly contribute toward mass flexion-extension co-excitation patterns during walking. The current study sought to investigate the associations between damage to corticospinal (CST) and corticoreticular (CRP) motor pathways following stroke and the presence of mass flexion-extension patterns during walking as evaluated using module analysis.

**Methods:**

Seventeen healthy controls and 44 stroke survivors were included in the study. We used non-negative matrix factorization for module analysis of paretic leg electromyographic activity. We typically have observed four modules during walking in healthy individuals. Stroke survivors often have less independently timed modules, for example two-modules presented as mass flexion-extension pattern. We used diffusion tensor imaging-based analysis where streamlines connecting regions of interest between the cortex and brainstem were computed to evaluate CST and CRP integrity. We also used a coarse classification tree analysis to evaluate the relative CST and CRP contribution toward module control.

**Results:**

Interhemispheric CST asymmetry was associated with worse lower extremity Fugl-Meyer score (*p* = 0.023), propulsion symmetry (*p* = 0.016), and fewer modules (*p* = 0.028). Interhemispheric CRP asymmetry was associated with worse lower extremity Fugl-Meyer score (*p* = 0.009), Dynamic gait index (*p* = 0.035), Six-minute walk test (*p* = 0.020), Berg balance scale (*p* = 0.048), self-selected walking speed (*p* = 0.041), and propulsion symmetry (*p* = 0.001). The classification tree model reveled that substantial ipsilesional CRP or CST damage leads to a two-module pattern and poor walking ability with a trend toward increased compensatory contralesional CRP based control.

**Conclusion:**

Both CST and CRP are involved with control of modules during walking and damage to both may lead to greater reliance on the contralesional CRP, which may contribute to a two-module pattern and be associated with worse walking performance.

## Introduction

The post-stroke loss of full ability to independently activate groups of muscles from a mass flexion or extension co-excitation pattern, typically referred to as an “abnormal flexion or extension muscle synergy” by clinicians (1, 2), often limits functional walking ability after stroke (3). The Fugl-Meyer Assessment (FMA) (4) is the current gold standard measure of motor impairment post-stroke. FMA was developed to assess a person's progressive ability to move outside of flexor or extensor synergies, based on the original work on stages of motor recovery presented by Twitchell and Brunnstrom (4). Specifically, in this framework the most severely affected stroke survivors are believed to be dependent on the flexor or extensor synergies and only have the ability to produce a stereotypical mass flexion or extension co-excitation pattern. Although, previous literature has consistently demonstrated correlations of FMA with walking performance (5–7), the FMA has limitations in evaluating lower extremity motor impairments as an indicator of ability to move outside of mass flexion or extension co-excitation during walking because it assesses motor impairments in the context of performance of discrete volitional tasks upon command and not during walking (5). Previous work from our lab has shown that co-excitation of independent muscle groups or normal muscle synergies that are associated with specific biomechanical functions, can explain motor coordination during walking (8, 9). We refer to these synergies as modules to avoid confusion with the clinical definition of flexor or extensor synergies. A reduction in the number of independent modules used by a person occurs following stroke as a result of lesser independently timed muscle activity i.e., merging of modules (3, 10). For example, healthy individuals often use four modules during walking, but stroke survivors may present with two or three from merging of modules (3, 10). Reduced number of modules result in poorly executed biomechanical functions and hence poor walking ability (3, 11). These modules can evaluate motor coordination during post-stroke walking more appropriately than the FMA because number of modules correlates more strongly with a wide range of walking performance measures than does FMA (5). Establishing a better understanding of the relationships between the health of the underlying neural pathways, the reduced number of independent modules and the associated gait disturbances following stroke will likely facilitate identifying appropriate approaches for enhancing motor recovery and restoring gait function in stroke survivors.

The corticospinal tract (CST) is the major descending motor pathway involved in control of independent muscle activation in skilled movements (12), but its role in producing either the independent modules in neurologically healthy walking or the reduced number of modules often seen during post-stroke walking may be limited, as it is known that non-CST pathways and spinal interneuronal networks also contribute to the control of muscle activity during walking that is quantified by the modules (13, 14). Despite the contributions of other neural pathways and structures, evidence suggests that CST is involved in control of muscle activity during walking in individuals without a neurological injury (15–17). Furthermore, animal studies have demonstrated involvement of cortical control in regulating modules during reaching and walking tasks (12, 18, 19). Activation of the pyramidal tract neurons that are the origin of the CST is associated with modulating activity of small groups of muscles at different times of the gait cycle in cats (12, 18). Therefore, descending commands from an intact CST may shape module activation patterns during walking. Research in human participants has shown that a sub-population of stroke survivors are able to walk following complete unilateral CST damage (20–22). Therefore, it appears that the CST may not be necessary to control steady-state walking (20, 23). But it is not known whether the full range of independently timed modules seen during the gait cycle of neurologically intact persons would still be present without CST involvement. It is likely that activation of muscle modules (i.e., locomotor coordination) is an interplay of CST and non-CST pathways, as well as brainstem and spinal networks (13, 14). Drew et al. (24) proposed that the corticoreticular-reticulospinal-spinal interneuronal (CRSI) network is responsible for a general motor pattern during walking that is augmented by CST tracts for modification of muscle activity specific to the task demands. Thus, we expect non-CST pathways and spinal networks may also have substantial involvement in producing muscle activity following stroke, although the modules produced by such pathways may not be the same as those used for walking by neurologically intact persons (i.e., they may be a merged combination of the intact modules).

The corticoreticular pathway (CRP) is one of the non-CST or extra-pyramidal pathways involved with normal locomotor control and recovery following a neurological injury and given that it coactivates multiple muscles bilaterally it has the potential to play a role in the expression of mass flexion and extension co-excitation of modules during walking (24). In the stroke population, Jang et al. (22). demonstrated that 34 of 54 individuals with a complete unilateral CST injury (determined *via* diffusion tensor tractography) were able to walk independently. Out of the 34 independent walkers, four subjects had an intact CRP in the affected hemisphere. Independent walkers also had significantly greater CRP fiber volume in the unaffected hemisphere than healthy individuals or stroke survivors who could not walk independently. These observations suggest possible compensation of contralesional CRP demonstrated as increased connectivity in those with complete CST damage, which may then be associated with recovery of walking. Individuals with severe motor impairment after stroke show that unilateral pedaling by the non-paretic leg evokes rhythmic muscle activity in a resting paretic leg that is of a magnitude similar to that seen during bilateral pedaling (25). This is consistent with increase in the expression of contralesional CRP control following decreased ipsilesional CST and CRP drive. As part of the CRSI network, CRP is involved with control of motor function of the trunk and legs (24, 26–28). Animal research shows that the CRSI network has bilateral projections at brainstem and spinal cord levels and modulates the flexor and extensor muscle coordination at different phases of a gait cycle during steady state walking as well as during challenging walking tasks (26, 29–31). Research in human participants has shown that severely affected stroke survivors often present with a two-module pattern. This pattern includes one module that is active during stance typically including activity of hamstrings, hip and knee extensors, and plantarflexors (all of which are typically represented in separate modules in the neurologically healthy), and a second module active during swing that includes primarily dorsiflexor activity, as well as additional activity in rectus femoris and hamstrings (3). Therefore, after damage to CST there may be an increased expression of the ipsilateral and/or contralateral CRP and/or SI network which could lead to fewer independent modules and more reliance on alternating flexion-extension patterns, i.e., a two-module pattern.

The purpose of the current study was to investigate the relationship between the use of a two-module flexion-extension pattern during walking and motor pathway integrity (both CST and CRP bilaterally) as a consequence of damage and increased compensatory connectivity due to a stroke lesion. We hypothesized that increased damage to ipsilesional CST or both ipsilesional CST and ipsilesional CRP, leads to more dependence upon contralesional CRP that would produce a less fractionated two-module pattern (a mass flexion-extension co-excitation pattern) of the paretic leg and poor walking ability. Establishing the relationships between motor pathway integrity and fewer independently timed modules used by post-stroke individuals during walking will improve our underlying models of hemiparetic motor control during walking and allow us to better inform rehabilitation approaches that can target the individual's motor control issues (i.e., identifying responders and non-responders for contralesional inhibitory or excitatory neuromodulation approaches).

## Methods

### Subjects

The research database registry of the Center of Biomedical Research Excellence in Stroke Recovery (IRB approved for data sharing) was queried for all participants with chronic stroke (>6 months) for whom we had neuroimaging, clinical, kinetic, and electromyography (EMG) data collected during walking from various IRB approved studies. Fourty-four stroke survivors were included in this convenience sample. Neuroimaging data for seventeen similarly-aged healthy individuals were also included. All subjects signed a written informed consent approved by the institutional review board at Medical University of South Carolina to have their data included in the database. All assessments were done by a team of licensed physical therapists and study staff.

### Clinical and biomechanical data acquisition

Clinical assessments included the lower extremity Fugl-Meyer (FMA-LE), Berg Balance Test (BBS), Dynamic Gait Index (DGI) and six-minute walk test (SMWT). Participants completed three trials of walking across a GaitRite (CIR Systems, Inc.; Franklin, NJ) to determine overground walking speed and three 30 s trials of walking on a split belt instrumented treadmill (Bertec Instrumented treadmill; Columbus, OH) at their self-selected walking speed to collect neuromechanical data. Ground reaction force (GRF) data sampled at 2,000 Hz were used to define gait events. Surface EMG data were recorded (Motion Lab Systems; Baton Rouge, LN, USA) from the following eight muscles bilaterally during treadmill walking assessments: tibialis anterior (TA), soleus (SO), medial gastrocnemius (MG), vastus medialis (VM), rectus femoris (RF), medial hamstrings (MH), lateral hamstrings (LH) and gluteus medius (GM). However, only data from the paretic leg are reported for the current analysis. EMG sampling rate was set at 2,000 Hz. Our previous work has determined that treadmill walking was appropriate for studying propulsion generation (32) and other neuromechanical measures of motor control deficits (33) after stroke.

### Biomechanical data analysis

Ground reaction force data from the instrumented treadmill were used to calculate Paretic propulsion (Pp) and Paretic Step Ratio (PSR). Pp is a quantitative measurement of the coordinated output of the paretic leg in comparison to the non-paretic leg for creating a forward propulsive force during walking. Pp was derived by dividing the positive anterior impulse from the paretic leg with the sum of positive anterior impulse from both legs (34). The positive anterior impulse is the time integral of the anterior GRF sampled during walking on the treadmill and is that component which accelerates the body forward. PSR is the percentage of stride length that is completed by the paretic leg and is used to quantify step length asymmetries. PSR is calculated by dividing the paretic step length by the stride length (35). We then derived the deviation from symmetry of Pp and PSR by subtracting Pp and PSR values from 0.5 and taking the absolute value. Therefore, values closer to 0 would represent greater symmetry in both cases.

### Module-based analysis

Electromyography data were high-pass filtered (40 Hz) with zero lag fourth-order Butterworth filter, demeaned, rectified and low-pass filtered (4 Hz) with zero lag fourth-order Butterworth filter. EMG amplitude for each muscle was averaged within each bin (or region of the gait cycle: first double support, first half of ipsilateral single leg stance, second half of ipsilateral single leg stance, second double support, first half of ipsilateral swing, second half of ipsilateral swing) during self-selected treadmill walking. The average value of the bin with the highest average was used to normalize EMG amplitude across all trials for an individual. This method of normalization was used because normalizing to the mean amplitude during the activity of interest rather than peak amplitude has been reported as a better method in reducing variability between subjects (36, 37). The EMG data were then time normalized to the gait cycle (0–100%). Average of the time normalized gait cycles were used for further analysis. Nonnegative matrix factorization (NNMF) was used to extract modules from the paretic leg for each participant using post-processed EMG signals (3). NNMF was used to calculate a variability accounted for (VAF) for each muscle and a cumulative VAF for all muscles in each bin. Modules were added until VAF for all muscles and bins of the gait cycle were ≥90% or until adding a module did not increase VAF >5% in any individual muscle or gait cycle bin (3). All EMG post-processing was done in MATLAB 8 (Mathworks, Inc.; Natick, MA, USA).

### MRI data acquisition

The MRI examination for all subjects included standard anatomical images and diffusion tensor imaging (DTI). Images were acquired using a 3T Siemens Trio or Prisma scanner (Siemens Healthcare, Erlangen, Germany) with a 12-channel head coil. A whole brain T1-weighted magnetization-prepared rapid acquisition gradient-echo (MPRAGE) sequence (TR: 1,900 ms, TE: 2.26 ms, T1: 900 ms, acquired isotropic voxel size: 1 mm × 1 mm × 1 mm, SNR: 1), T2-weighted FLAIR (TR: 9,000 ms, TE: 93 ms, T1: 2,500 ms, acquired voxel size: 0.9 mm × 0.9 mm × 4 mm, SNR: 1), and Diffusion weighted images were acquired (TR: 6,400 ms, TE: 96 ms, acquired isotropic voxel size: 2.7 mm × 2.7 mm × 2.7 mm, SNR: 1, *b* = 0, 1,000, and 2,000 s/mm^2^).

### Image processing

#### Lesion masks

Brain lesions for stroke survivors were manually traced on the T2-weighted FLAIR using MRIcron under the guidance of a neurologist (Bonilha). The stroke lesion maps were spatially normalized to standard space using SPM12 (Functional Imaging Laboratory, Wellcome Trust Center for Neuroimaging Institute of Neurology, University College London; http://www.fil.ion.ucl.ac.uk/spm/software/spm12/) and MATLAB scripts developed inhouse (38). In the first step, the T2 scan was co-registered with the individual's T1 scan, and the transforms were used to reslice the lesion map into native T1 space. Next, the resliced lesion map was smoothed with a 3 mm full-width half maximum Gaussian kernel to remove jagged edges associated with manual drawing. The smoothed lesion map was then binarized (lesioned vs. non-lesioned tissue) with a threshold of zero. SPM12's unified segmentation-normalization (39) for an enantiomorphic normalization approach (40) was applied to normalize the T1-weighted images onto the standard space. For this, we used a chimeric T1-weighted image where the area corresponding to the stroke lesion was replaced by the mirrored equivalent region in the intact hemisphere. The volume of the overall stroke lesion was calculated based on the number of lesioned voxels.

#### Tracing of sub-cortical regions of interest

As described in previous studies (22, 28, 41–43), the region of interest (ROI) to track the CST was manually traced in the pyramid of the medulla and the ROI to track the CRP was manually traced in the reticular formation of the medulla on each individual's B0 diffusion image using MRIcron.

### Structural connectome

We used a previously published connectome-based streamline approach to identify cortical and sub-cortical connectivity (44, 45). Each participant's individual connectome was built from structural and diffusion weighted neuroimaging data. T1 weighted images in standard space were segmented into probabilistic gray and white matter maps using SPM12's unified segmentation-normalization. Each individual's gray matter map was divided into 384 regions using the Atlas of Intrinsic Connectivity of Homotopic Areas (AICHA) brain atlas. The gray matter parcellation maps were non-linearly registered into the DTI space. The manually traced subcortical ROIs were then merged with specific cortical ROIs selected from the AICHA atlas in DTI space, creating individualized atlases (henceforth referred to as the AICHA-hybrid brain atlas) consisting of cortical and subcortical regions of interest. Diffusion images were undistorted using Eddy (46), and pairwise probabilistic DTI fiber tracking was performed. Tractography was estimated using FSL's FMRIB's Diffusion Toolbox (FDT) probabilistic method (47) with FDT's accelerated BEDPOST (48) used to assess default distributions of diffusion parameters at each voxel. Specifically, tractography was performed using FDT's probtrackX (parameters: 5,000 individual pathways drawn through the probability distributions on principal fiber direction, curvature threshold set at 0.2, 200 maximum steps, step length 0.5 mm, and distance correction) and streamlines connecting ROIs were computed. The number of streamlines connecting each pair of ROIs was corrected for the distance and size to compensate for the distance between ROIs and unequal size of gray matter ROIs respectively. The weighted CST connections were then determined by selecting streamlines passing through the seed ROI in the pyramidal tract portion of the medulla and cortical regions of the AICHA-hybrid brain atlas corresponding to Brodmann area 4. The weighted CRP fibers were determined by placing the seed ROI in the reticular formation of the medulla and cortical regions of the AICHA-hybrid brain atlas corresponding to Brodmann area 6 (41). Inter-hemispheric asymmetry for CST and CRP was computed as the ratios of CST and CRP streamlines respectively between the two hemispheres.

Inter-hemispheric asymmetry in healthy controls:


(1) $$\begin{array}{l} Assym_{Healthy}=\left(\frac{Streamline\;from\;right\;hemisphere\;}{Streamline\;from\;left\;hemisphere}\right) \end{array}$$

Inter-hemispheric asymmetry in stroke survivors:


(2) $$\begin{array}{l} Assym_{Stroke}=\left(\frac{Streamline\;from\;lesioned\;hemisphere}{Streamline\;from\;non-lesioned\;hemisphere}\right) \end{array}$$

Inter-hemispheric asymmetry value of 1 would indicate an equal number of streamlines in both hemispheres, a value of >1 would suggest more streamlines of right/lesioned hemisphere and a value <1 would mean more streamlines of left/non-lesioned hemisphere. Previous literature has shown that a leftward or rightward asymmetry is observed in healthy individuals with no neurological disorders (45). Therefore, we believe that inter-hemispheric streamline ratio is a better representation of asymmetry in comparison to a matrix that would assume a perfect symmetry between hemispheres and normalize by the sum of streamlines in each hemisphere.

### Voxel-based lesion symptom mapping

Voxel-based lesion symptom mapping (VLSM) (49) was performed to define the relationship between lesion location and number of modules where lesioned voxels were the independent variables. Based on previous literature only voxels lesioned in ≥10% of the patients were included (50–52), i.e., more than 4 individuals in the current study. We used the NiiStat toolbox in MATLAB version R2019b and SPM12 to perform the lesion symptom mapping analyses. All right sided lesions were flipped to the left hemisphere for VSLM.

### Statistical analysis

To control for the potential effect of scanner type (the scanner was upgraded during the time of data collection), connectivity strengths were harmonized by applying ComBat (53, 54). Although, such an adjustment allows us to increase our statistical power by integrating data from the two scanners it may result in negative values for smaller connectivity strengths. Mann-Whitney test was used to identify differences in inter-hemispheric asymmetry between healthy controls (Asymm_Healthy_) and stroke survivors (Asymm_Stroke_) for CST and CRP streamlines. Spearman's correlation was used to identify the relationship between modules and Asymm_Stroke_ for CST and CRP. Spearman's correlation was also used to explore the relationship of CST and CRP Asymm_Stroke_ with our secondary outcome measures of stroke survivors' walking ability, including self-selected over-ground walking speed, FMA-LE, BBS, DGI, SMWT, Pp, and PSR. In all cases, significance was set at *p* < 0.05. We did not adjust for multiple comparisons because the walking ability measures are interrelated and not independent comparisons. We believe that the associations of secondary outcome measures with motor pathways can provide useful exploratory data for future dedicated studies on these measures to confirm the results. For VSLM one-tailed statistical tests were applied based on the assumption that lesioned tissue will lead to impairment (fewer modules).

To further understand the relationship between CST and CRP asymmetry in producing two module patterns, a Coarse Classification tree algorithm with five-fold cross validation in MATLAB was used to classify the module categories with Asymm_Stroke_ for CST and CRP as predictor variables. Although, this particular model will need validation, the preliminary findings can be a useful direction for future research to establish biomarkers and neuromodulation targets to improve mass flexion-extension patterns. Because we wanted to evaluate the role of motor pathway integrity in generating mass flexion-extension co-excitation patterns, we dichotomized the stroke survivors into either individuals with two modules or individuals with more than two modules (three or four) for both the classification tree model and lesion mapping. To further, understand the characteristics of correctly classified individuals with more than two modules (groupA_Correct>2Modules_), incorrectly classified individuals with two modules (groupB_Incorrect2Modules_), and correctly classified individuals with two modules (groupC_Correct2Modules_), we performed a *post-hoc* analysis to evaluate differences in their walking ability and CRP and CST streamlines using One-way ANOVA.

## Results

**Figure 1:**
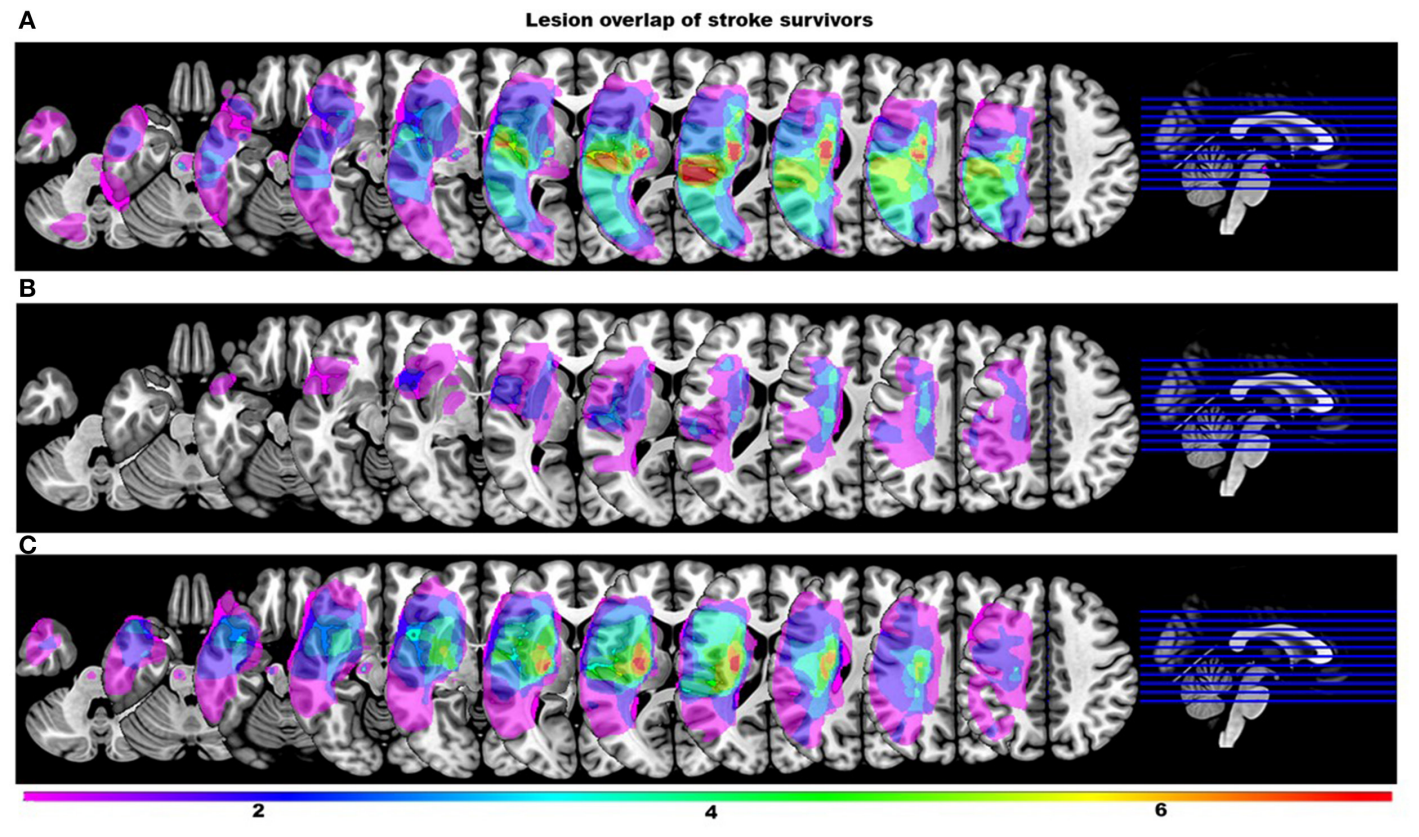
Lesion overlay maps of stroke survivors stratified by the number of modules. Color scale indicates the number of stroke survivors having a lesion in every voxel (all right sided lesions are flipped to the left hemisphere for clarity). **(A)** Overlay maps of stroke survivors with more than two modules. **(B)** Overlay maps of stroke survivors with two modules that were incorrectly classified by the classification tree as individuals with more than two modules. **(C)** Overlay maps of stroke survivors with two modules correctly classified by the classification tree.

One stroke survivor's streamline data were extreme outliers and could not be explained by the typical variability. Upon further investigation they were excluded from the analysis due to lack of clear evidence of a liquefactive necrosis on the T2-weighted image following a single stroke. All remaining stroke survivors had a lesion in supratentorial/infratentorial regions. Figure 1 shows a lesion overlay of stroke survivors dichotomized by their use of a mass flexion-extension co-excitation patterns (i.e., either two or more than two modules). Participant demographic data for both groups are presented in Table 1.

**Table 1:** Summary data for stroke participant demographics.

**Sample size (*n*)**	**44**
Age (years)	61.8 (11.2)
**Sex**
Female	14 (32%)
Male	30 (68%)
**Lesion side**
Right	18 (41%)
Left	26 (59%)
Months post-stroke	50.5 (48.8)
FMA-LE	26.3 (5.03)

Continuous variables are presented as mean (standard deviation).

Categorical variables are presented as a frequency count (percentage).

**Figure 2:**
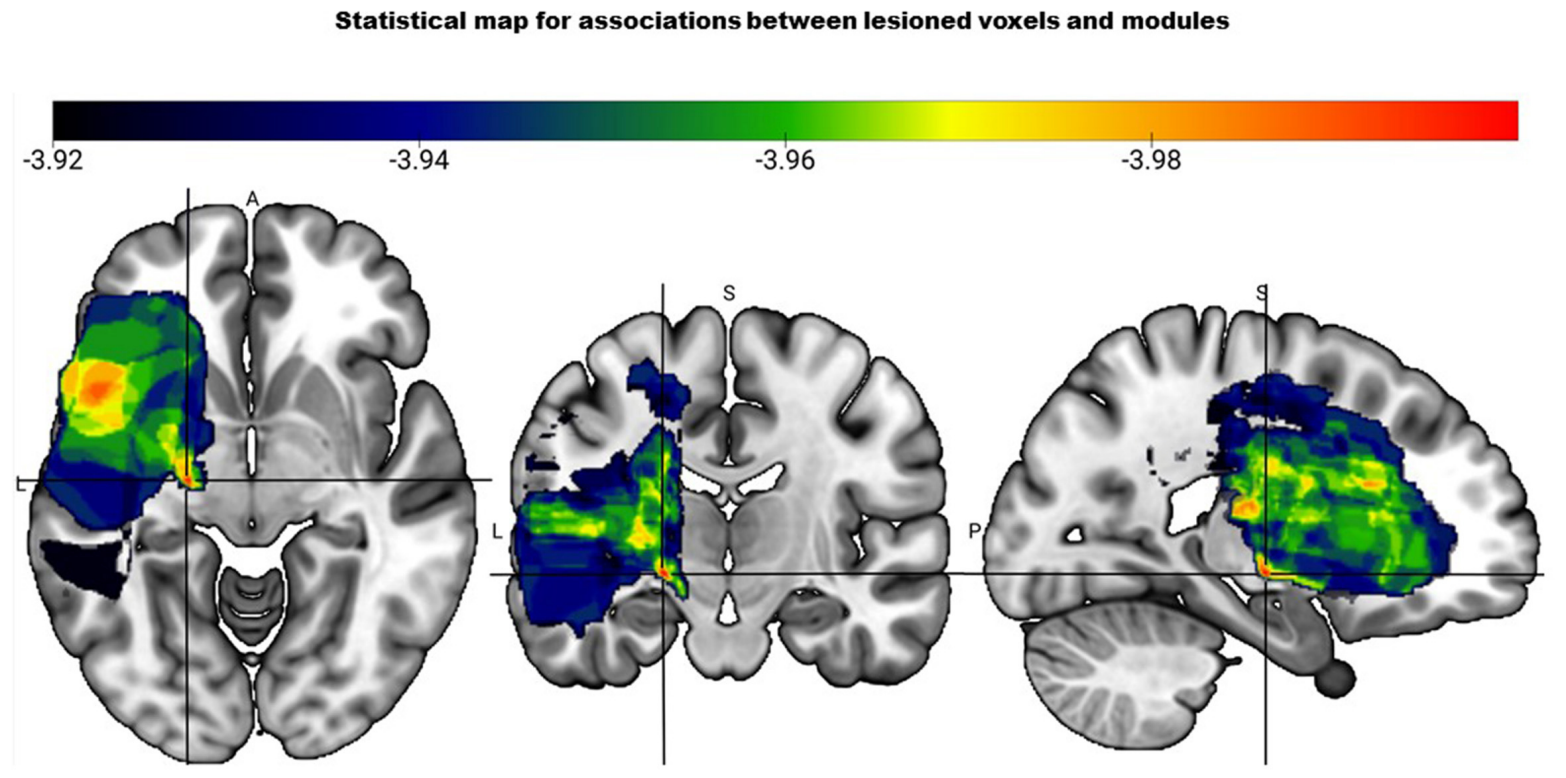
Statistical map based on the voxel-based lesion mapping analysis shows the association between number of modules and lesioned voxels. Color scale indicates strength of association between two module pattern and lesion location with red having the strongest association (represented by the crosshair).

VSLM analysis revealed that one voxel survived the corrected threshold of *z* <-3.96. This voxel was in the cerebral peduncle that contains CST, suggesting significant association between CST lesion within the cerebral peduncle and a two-module pattern (Figure 2). However, the data should be interpreted with caution as only one out of 262,481 voxels survived the threshold.

### Recovery of walking ability is significantly dependent on inter-hemispheric asymmetry of CST and CRP motor pathways

**Figure 3:**
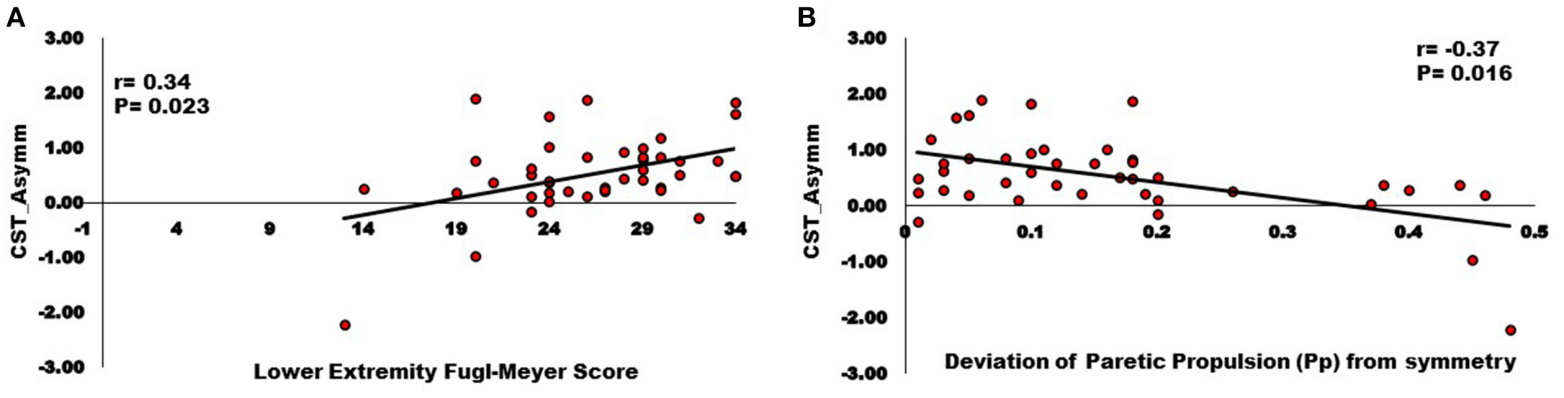
Relationships between Inter-hemispheric asymmetry of CST streamlines and **(A)** Lower extremity Fugl-Meyer score; **(B)** Deviation of paretic propulsion (Pp) from symmetry. The Asymm_Stroke_ for CST (i.e., fewer CST streamlines on the lesioned hemisphere) were positively correlated with Lower extremity Fugl-Meyer score, and negatively correlated with deviation from symmetric Pp (*p* < 0.05).

**Figure 4:**
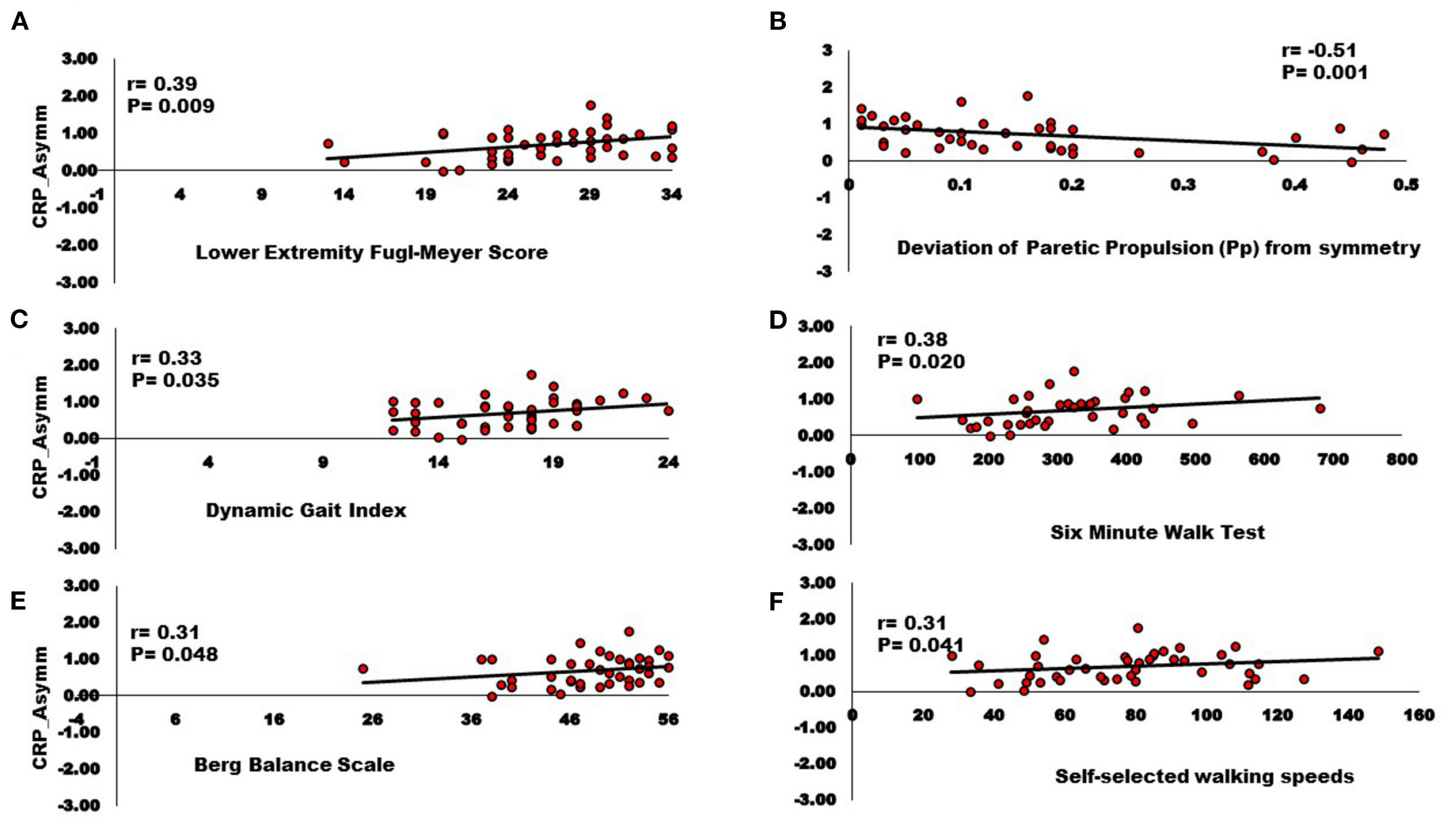
Relationships between Inter-hemispheric asymmetry of CRP streamlines and **(A)** Lower extremity Fugl-Meyer score; **(B)** Deviation of paretic propulsion (Pp) from symmetry; **(C)** Dynamic gait index; **(D)** Six-minute walk test; **(E)** Berg balance scale; **(F)** Self-selected walking speed. The Asymm_Stroke_ for CRP (i.e., fewer CRP streamlines on the lesioned hemisphere) were positively correlated with Lower extremity Fugl-Meyer score, Dynamic gait index, Six-minute walk test, Berg balance scale, Self-selected walking speeds, and negatively correlated with deviation from symmetric Pp (*p* < 0.05).

**Figure 5:**
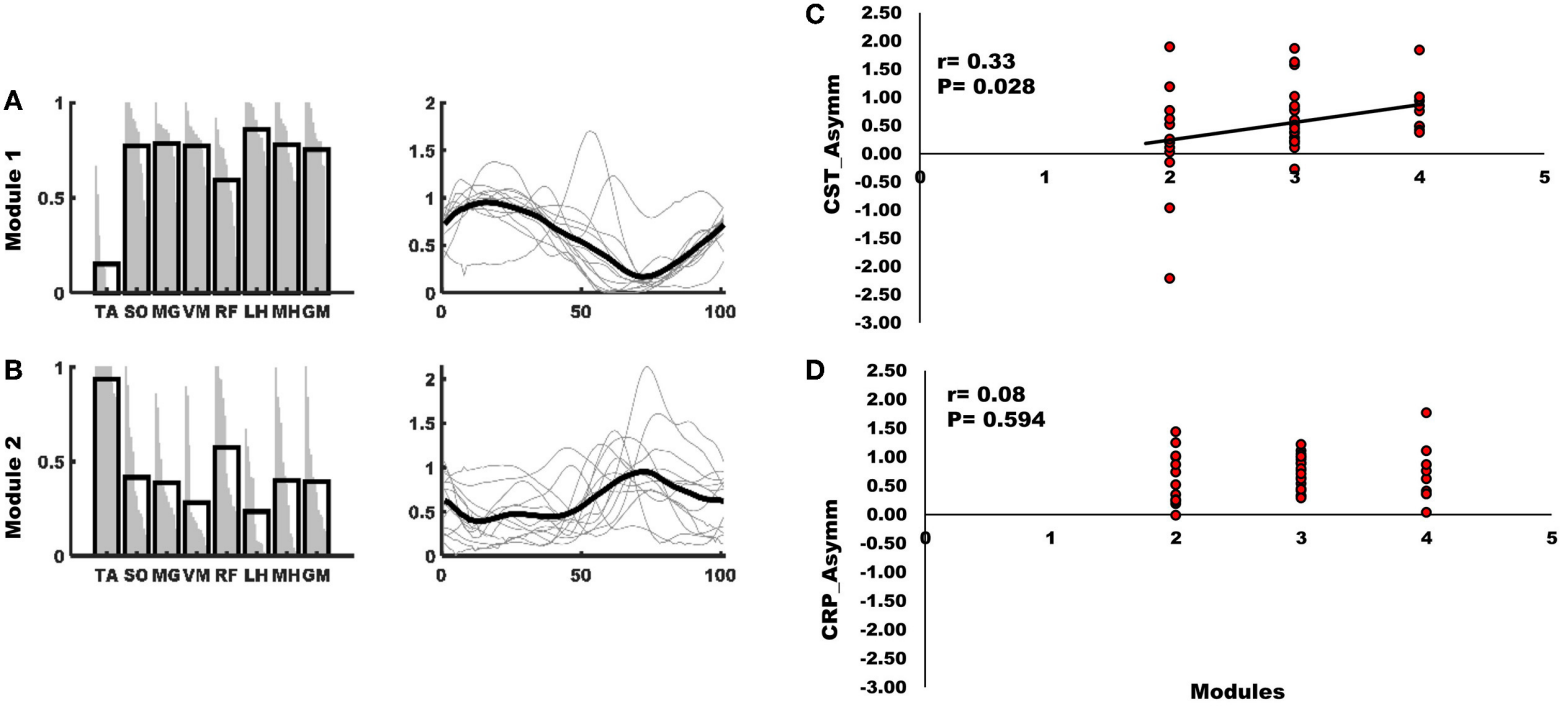
**(A)** Module one of the mass flexion-extension pattern had greater activity of SO, MG, VM, LH, MH, and GM and was primarily active during stance. **(B)** The second module of the mass flexion-extension pattern had greater activity of TA and RF and was primarily active during swing. Each black bar on the left panel of **(A,B)** indicates group mean of the representation of a muscle within the module and each gray line within the black bar represents a subject. Black line on the right panel of A and B indicates the group mean and gray lines represent the activity profile of the module for each subject. **(C)** The Asymm_Stroke_ for CST (i.e., fewer CST streamlines on the lesioned hemisphere) were positively correlated with number of modules. **(D)** The correlation between Asymm_Stroke_ for CRP (i.e., fewer CRP streamlines on the lesioned hemisphere), and number of modules was not statistically significant (*p* < 0.05).

Correlation analyses support associations of motor pathway integrity with altered modules and walking ability. There was significantly larger interhemispheric asymmetry (Asymm_Stroke_ = Lesioned/Non-lesioned; Asymm_Healthy_ = Right/Left) of CST and CRP streamlines in the stroke population compared to healthy individuals (CST for Asymm_Healthy_:0.96, Asymm_Stroke_:0.53, *p* = 0.010), (CRP for Asymm_Healthy_:0.91, Asymm_Stroke_: 0.69, *p* = 0.040). Since the research database registry of the COBRE in Stroke Recovery was used to aggregate data from several individual studies for the current study, not all outcome measures were available for each subject. We found a positive correlation between Asymm_Stroke_ for CST and the number of modules (*df* = 41, *r* = 0.33, *p* = 0.028), FMA-LE (*df* = 41, *r* = 0.34, *p* = 0.023), as well as negative correlation with deviation from symmetric Pp (*df* = 39, *r* = −0.37, *p* = 0.016; Figure 3). Asymm_Stroke_ for CRP were positively correlated with FMA-LE (*df* = 41, *r* = 0.39, *p* = 0.009), DGI (*df* = 40, *r* = 0.33, *p* = 0.035), SMWT (*df* = 36, *r* = 0.38, *p* = 0.020), BBS (*df* = 40, *r* = 0.31, *p* = 0.048), self-selected walking speed (*df* = 41, *r* = 0.31, *p* = 0.041), and negatively correlated with deviation from symmetric Pp (*df* = 39, *r* = −0.51, *p* = 0.001; Figure 4). There were no significant correlations between Asymm_Stroke_ for CST and self-selected walking speed (*df* = 41, *r* = 0.29, *p* = 0.055), DGI (*df* = 40, *r* = 0.17, *p* = 0.287), SMWT (*df* = 36, *r* = 0.20, *p* = 0.216), BBS (*df* = 40, *r* = 0.18, *p* = 0.265) and deviation from symmetric PSR (*df* = 37, *r* = −0.02, *p* = 0.921; Table 2). Additionally, no significant correlations were seen between Asymm_Stroke_ for CRP and number of modules (*df* = 41, *r* = 0.08, *p* = 0.594; Figure 5) and deviation from symmetric PSR (*df* = 37, *r* = −0.03, *p* = 0.831; Table 2).

**Table 2:** Correlations of motor pathways with biomechanical and clinical measures.

	**DGI**	**FMA_LE**	**SMWT**	**BBS**	**Pp_deviation_**	**PSR**	**SS**
**Asymm**_**Stroke**_ **CST**	*r* = 0.17	*r* = 0.34	*r* = 0.20	*r* = 0.18	*r* = −0.37	*r* = −0.02	*r* = 0.29
	*p* = 0.287	*p* = 0.023^*^	*p* = 0.216	*p* = 0.265	*p* = 0.016^*^	*p* = 0.921	*p* = 0.055
**Asymm**_**Stroke**_ **CRP**	*r* = 0.33	*r* = 0.39	*r* = 0.38	*r* = 0.31	*r* = −0.51	*r* = −0.03	*r* = 0.31
	*p* = 0.035^^*^^	*p* <0.009^^*^^	*p* = 0.020^^*^^	*p* = 0.048^^*^^	*p* = 0.001^^*^^	*p* = 0.831	*p* = 0.041^^*^^

* Indicate significant correlations (*p* < 0.05).

### Impaired walking ability is associated with fewer modules

Consistent with previous literature NNMF based analysis revealed a two, three, or four modules pattern in individuals with stroke (3, 5, 10, 55, 56). We also found that number of modules had a significant positive correlation with several clinical outcome measures, effectively reproducing some of the results of previous published relationships (3) on a second large dataset. A positive correlation was seen between number of modules and FMA-LE (*df* = 41, *r* = 0.55, *p* < 0.001), DGI (*df* = 40, *r* = 0.32, *p* = 0.039), and BBS (*df* = 40, *r* = 0.37, *p* = 0.017). However, we did not see any significant correlation between number of modules and deviation from symmetric Pp (*df* = 39, *r* = −0.13, *p* = 0.419), deviation from symmetric PSR (*df* = 37, *r* = 0.07, *p* = 0.687), SMWT (*df* = 36, *r* = 0.25, *p* = 0.125), and self-selected walking speed (*df* = 41, *r* = 0.25, *p* = 0.100).

### Stroke survivors with greater inter-hemispheric asymmetry of CST and CRP are likely to have mass flexion-extension co-excitation patterns

**Figure 6:**
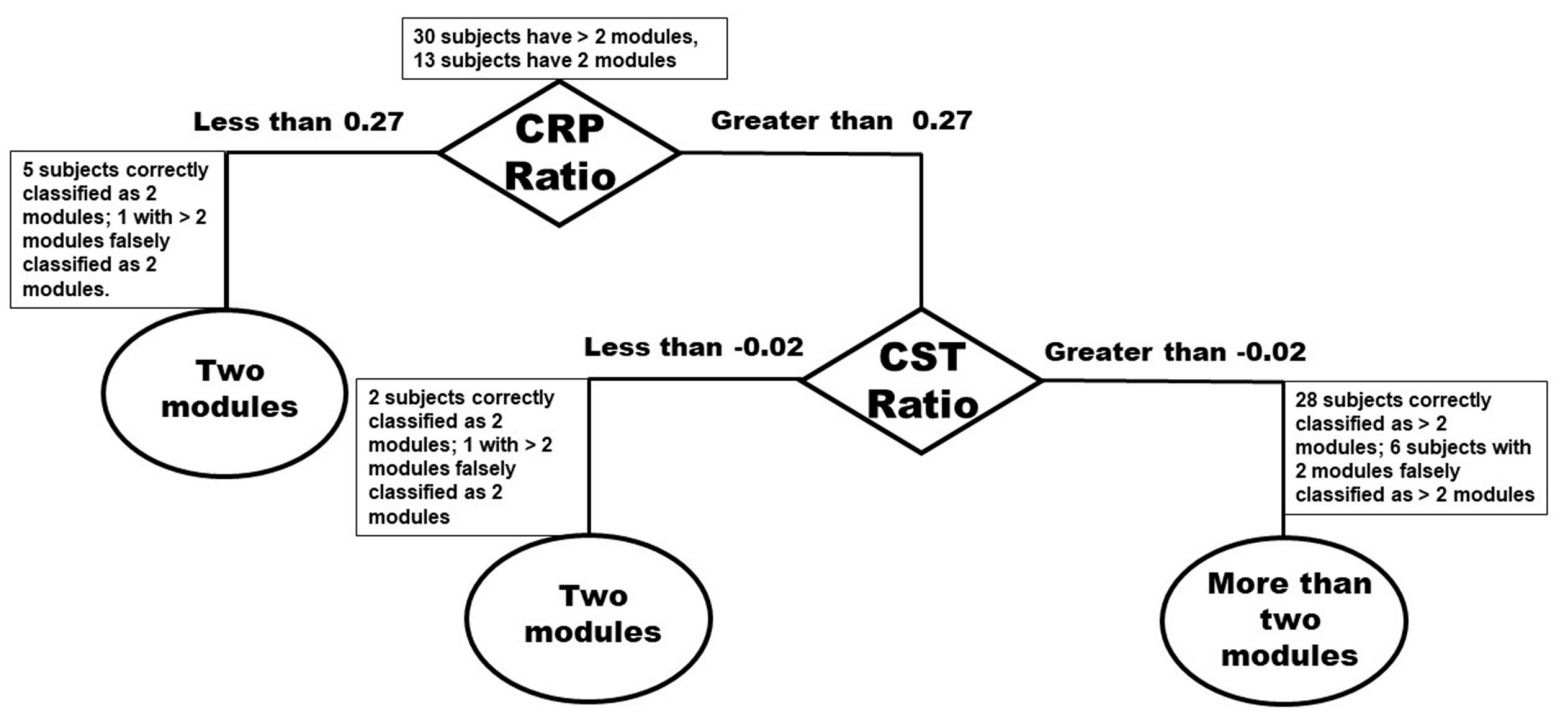
Classification tree based on number of modules and Inter-hemispheric asymmetry of CST and CRP streamlines.

Classification tree analysis suggests that individuals had a mass flexion-extension pattern of co-excitation (i.e., two modules) if they had either significant asymmetry of CRP or had more moderately asymmetric CRP but significant asymmetry of CST streamlines (Figure 6). Specifically, stroke survivors with asymmetry of CRP streamlines < 0.27 (Asymm_Healthy_ = 0.91) or asymmetry of CST streamlines <-0.02 (Asymm_Healthy_ = 0.96) were predicted to have two modules (Figure 6). The model correctly classified 28 of 30 individuals (93%) with more than two modules (i.e., groupA_Correct>2Modules_ defined as those who had CRP ratio >0.27 and CST ratio >-0.02 and were correctly classified as individuals with more than two modules). The model also correctly classified seven of 13 individuals (53%) with two modules (i.e., groupC_Correct2Modules_ defined as those who had CST ratio <-0.02 and/or CRP ratio <0.27 and were correctly classified as individuals with two modules). The model incorrectly classified eight individuals, six of whom had only two modules but were classified as more than

two modules (i.e., groupB_Incorrect2Modules_ defined as those individuals who had CRP ratio >0.27 and CST ratio >-0.02 and were incorrectly classified as individuals with two modules). The overall accuracy of the model was 81%.

**Figure 7:**
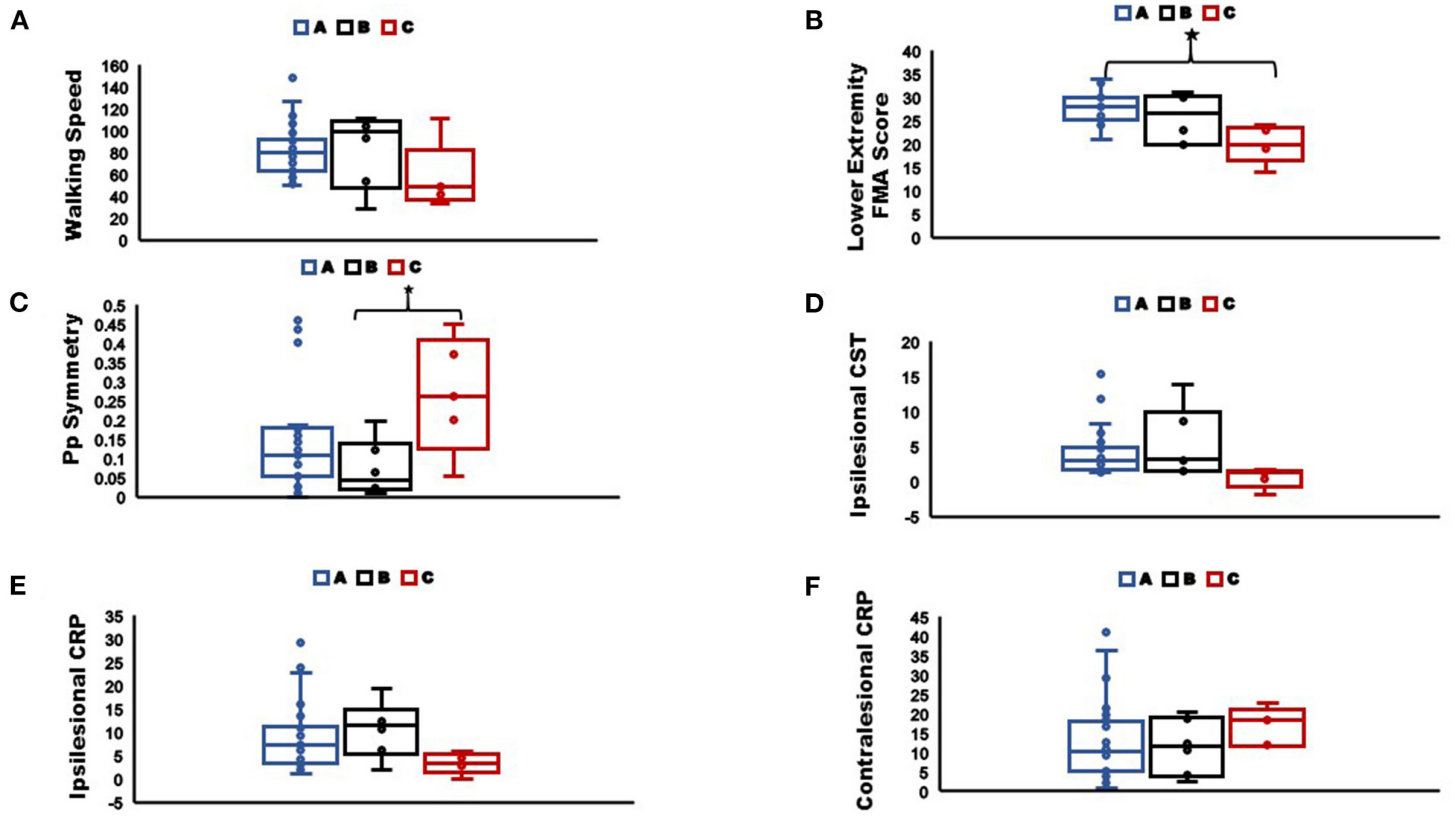
Comparisons of **(A)** Walking speed; **(B)** Lower extremity Fugl-Meyer score; **(C)** Pp symmetry; **(D)** Ipsilesional CST streamlines; **(E)** Ipsilesional CRP streamlines; **(F)** Contralesional CRP streamlines, between sub-groups as classified by the classification tree (*indicate significant difference between groups at *p* < 0.05). The box plots indicate the range in the data, horizontal line in the center is the median, the upper and lower boundaries of the box indicate the upper and lower quartile respectively, and markers represent the extreme values. Group A: correctly classified individuals with more than two modules, Group B: incorrectly classified individuals with two modules, Group C: correctly classified individuals with two modules.

*Post-hoc* analysis to characterize differences between correctly classified individuals with more than two modules (groupA_Correct>2Modules_), incorrectly classified individuals with two modules (groupB_Incorrect2Modules_), and correctly classified individuals with two modules (groupC_Correct2Modules_) revealed that groupC_Correct2Modules_ had most impaired walking ability compared to groupB_Incorrect2Modules_ and groupA_Correct>2Modules_. Specifically, GroupC_Correct2Modules_ (mean = 20) had significantly lower FMA-LE (*p* < 0.001) than groupA_Correct>2Modules_ (mean = 28.1) and a trend toward lower FMA-LE (*p* = 0.064) than groupB_Incorrect2Modules_ (mean = 25.6). We also found that groupC_Correct2Modules_ (mean = 0.26) were significantly less symmetric in Pp (*p* = 0.031) than groupB_Incorrect2Modules_ (mean = 0.07) and trended less symmetric in Pp than groupA_Correct>2Modules_ (mean = 0.14; *p* = 0.131). Although, not statistically significant we also found that individuals in groupC_Correct2Modules_ walked at slower speeds (mean = 0.57 m/s) than groupA_Correct>2Modules_ (mean = 0.82 m/s; *p* = 0.18) and groupB_Incorrect2Modules_ (mean = 0.83 m/s; *p* = 0.34; Figure 7). We also found that groupC_Correct2Modules_ had higher number of contralesional CRP streamlines (mean = 16.8) than groupA_Correct>2Modules_ (mean = 13.2; *p* = 1.0) and groupB_Incorrect2Modules_ (mean = 11.5; *p* = 1.0). GroupC_Correct2Modules_ also had lower ipsilesional CST streamlines (mean = 0.5) than groupA_Correct>2Modules_ (mean = 4.1; *p* = 0.14) and groupB_Incorrect2Modules_ (mean = 5.5; *p* = 0.79). Additionally, groupC_Correct2Modules_ had lower ipsilesional CRP streamlines (mean = 3.3) than groupA_Correct>2Modules_ (mean = 8.6; *p* = 0.30) and groupB_Incorrect2Modules_ (mean = 10.7; *p* = 0.23). Greater contralesional CRP streamlines in GroupC_Correct2Modules_ individuals is indicative of possible increased compensatory connectivity of contralesional CRP in response to fewer ipsilesional CST and CRP streamlines as has been shown in previous literature for individuals with complete CST lesions (22).

### FMA-LE based classification for number of modules may not work well for individuals with more than two modules

We also evaluated classification of modules based on the 22-point subsection of FMA-LE which assesses the ability to move out of flexor or extensor synergies to further understand the relationship between synergies and modules. FMA-LE is a reliable method (57) that is frequently used to evaluate (58, 59) or predict (6) motor function in stroke population. Therefore, we believe it is important to evaluate the performance of an FMA-LE based module classification in addition to the motor pathways based module classification to better understand the impact of the two measures. We used FMA-LE <15 as a cutoff for individuals likely to have two modules because these individuals are theoretically unable to move out of a mass flexion-extension co-excitation pattern and therefore would be expected to have a two-module pattern (5). This method correctly classified 10 out of 13 individuals with two modules (76%) and 24 out of 30 individuals with more than two modules (80%), for an overall accuracy of 79%. All misclassified individuals with two modules had moderately impaired walking ability. Thus, while the FMA-LE correctly identified a higher percentage of two module individuals (76 vs. 53%), it also had a higher false positive rate in misidentifying individuals with three or four modules as having two modules (19 vs. 6%).

## Discussion

In the current study, we investigated the relationship between walking using two modules and the integrity of motor pathways involved in locomotor control. We found that increased damage to both the CST and CRP were associated with walking using two modules. Although, previous literature in animal models and humans post-stroke has shown that steady state walking is possible without CST involvement, our results suggest that damage to the CST is associated with impaired motor coordination resulting in fewer independently timed modules and poorer walking performance. We found that CRP integrity was more strongly related to recovery of multiple clinical and biomechanical measures of walking ability than CST integrity. Although not statistically significant, we also found some evidence of increased contralesional CRP streamlines in a small subgroup of the most impaired individuals with two modules i.e., groupC_Correct2Modules_ (group mean = 16.8), compared to less impaired individuals with two modules i.e., groupB_Incorrect2Modules_ (group mean = 11.5), and also to individuals with more than two modules i.e., groupA_Correct>2Modules_ (group mean = 13.2; Figure 7). There is some indication that increased contralesional CRP may result in walking using two modules (i.e., less independence of usually independent modules from mass flexion-extension co-excitation pattern). Therefore, it appears that CST damage reduces the number of independent modules; furthermore, substantial ipsilesional CST and CRP damage may reveal an increased expression of contralesional CRP based control and result in impaired walking using only two modules.

Stroke survivors with greater inter-hemispheric CST asymmetry had fewer muscle modules and greater motor impairment. Stroke survivors had significantly greater inter-hemispheric asymmetry for CST streamlines in comparison to healthy individuals (CST Asymm_Stroke_ = 0.53 compared to CST Asymm_Healthy_ = 0.96), indicating fewer streamlines on the lesioned compared to non-lesioned hemisphere due to CST damage following a stroke lesion. Although theoretically there can be limited involvement of CST during steady-state walking, we found damage to CST is associated with worse FMA-LE scores, fewer modules, and poor propulsion symmetry suggesting that CST is a primary contributor to normal motor coordination during walking. Other studies with similar findings have shown that damage to CST leads to increased lower extremity motor impairment (60, 61), although those studies did not report on the number of modules used during walking. Our results support that CST damage is associated with a decreased ability to activate the full range of independent muscle modules normally used during steady state walking, which is likely a primary cause of poor walking ability.

We found that increased CRP asymmetry was also significantly associated with poorer walking ability and that CRP asymmetry provided the primary decision tree criteria for whether a survivor walked using two modules. Specifically, we found that increased inter-hemispheric asymmetry of CRP was significantly correlated with poorer walking ability. Additionally, a greater number of measures of walking ability were significantly associated with CRP asymmetry than were associated with CST asymmetry (Table 2). However, unlike with CST asymmetry we did not see a significant correlation between CRP asymmetry and modules (*r* = 0.08, *p* = 0.594), perhaps indicating less CRP involvement with partial independence from mass flexion or extension co-excitation patterns (i.e., those who walked with three modules instead of two). Animal models have suggested varying levels of CST and CRP involvement in fractionation of movements that depends on task demands (24, 62). The sub-cortical reticulospinal-spinal interneuronal network (i.e., target for CRP) coordinates mass flexion-extension co-excitation patterns and motor cortex (i.e., CST) provides additional control for more fractionated muscle activation patterns (63). More recently it was demonstrated that in healthy humans both CST and CRP are involved in upper extremity movement control and their relative contribution varies based on the complexity of tasks (64, 65). In stroke survivors, recovery of finger movements involves two network systems with one (presumably reticulospinal tracts) contributing predominantly to strength that represents a less fractionated muscle activation pattern and the second (presumably CST) providing additional fine motor control of finger movements (66). Our data appear to support a similar relationship for the lower extremity. The significant correlation between CST asymmetry and module number and lack of correlation between CRP asymmetry and module number could suggest that contralesional CRP produces a mass flexion-extension pattern with further fractionation of muscle activity into independent modules resulting predominantly from the CST.

We found evidence of increased compensatory connectivity of the contralesional CRP pathways in the most impaired stroke survivors. Stroke survivors had significantly greater inter-hemispheric asymmetry for CRP streamlines in comparison to healthy individuals (CRP Asymm_Stroke_ = 0.69; Asymm_Healthy_ = 0.91), indicating fewer streamlines on the lesioned compared to non-lesioned hemisphere, and those who were most impaired may have shown some increased compensatory connectivity of the contralesional CRP. Although this was not statistically significant, our *post-hoc* analysis indicated that individuals with two modules who had greater inter-hemispheric asymmetry for CST <-0.02 and/or CRP <0.27 (groupC_Correct2Modules_) had a trend toward more contralesional CRP streamlines than individuals with less asymmetric CRP and CST (Figure 7). Therefore, it is possible that significant increase in contralesional CRP connectivity (i.e., more streamlines) occurs only after severe ipsilesional CST and CRP damage. In studies of upper-extremity hemiparesis, greater impairment of reaching performance and exaggerated flexion synergy during shoulder abduction are associated with increased electroencephalography based contralesional activity in pre-motor cortex that is known to provide inputs to CRP (67). It has also been reported that increased contralesional CRP integrity at the brainstem and spinal cord level of stroke survivors is associated with lower upper-extremity FMA scores (68). Jang et al. (22) showed that stroke survivors who had a complete CST lesion had a positive correlation between their walking ability and increased compensatory connectivity of contralesional CRP. Furthermore, stroke survivors with a complete CST injury and inability to walk at the time of stroke are able to regain their walking ability following increased contralesional CRP connectivity (42). The nervous system prefers to rely on the undamaged tissue of the lesioned hemisphere following a stroke (69) with a secondary preference for involvement of the contralesional hemisphere (70). We speculate that following a complete CST lesion and possibly also much involvement of ipsilesional CRP, stroke survivors would mostly rely on contralesional CRP and would have likely walked using only two modules with poor walking ability. We believe that when ipsilesional CST and CRP pathways are more intact, there may be less dependence on contralesional CRP and hence little change in connectivity of the pathway.

In addition to the imaging-based CST and CRP classification, we also looked at the FMA-LE threshold-based classification for flexor or extensor synergies. We found that FMA-LE correctly identified a higher percentage of two module individuals compared to the motor pathways based classification tree, but it misidentified a higher percentage of individuals with three or four modules as having two modules. Therefore, when evaluating stroke survivors' ability to move outside of mass flexion-extension patterns, CST and CRP can provide us a better classification for less impaired individuals i.e., individuals with more than two modules, in comparison to FMA-LE that is often used by clinicians. We believe that evaluating the integrity of motor pathways is also informative and provides additional useful information to clinicians for developing personalized neuromodulation rehabilitation protocols.

This study provides novel information regarding the differential involvement of motor pathways in recovery of walking and muscle coordination that could have significant future implications for identifying a subject specific target for neuromodulation or identifying responders and non-responders to gait rehabilitation protocols focused on improving muscle coordination. However, some methodological considerations should be discussed. Limitations in diffusion tractography such as difficulty in estimating crossing fibers should be considered when interpreting our results. Additionally, limited resolution of the B0 image for anatomical tracing of brainstem regions of interest for tractography may have led to some false or missing CST and CRP streamlines. While lesion location can affect motor function and/or recovery and it varied across subjects (supratentorial/infratentorial), our focus on including the number of ipsilesional as well as contralesional streamlines of motorpathways corrected for the distance between ROIs and unequal size of gray matter ROIs, offsets some concerns of heterogeneity in the lesion location. Nevertheless, future studies with a larger sample size will better establish the relationship between lesion location and modules. A previous study has demonstrated that the EMG pre-processing methods and number of gait cycles included in the NNMF analysis may influence the modules extracted from the data (71). As suggested by Oliveira et al. (71) we have included at least 20 gait cycles for each subject's NNMF analysis. Additionally, based on extensive research form our lab (3, 5, 33) we have used robust criteria to identify the number of modules that accounts for variability across muscles as well as across each of the six bins of gait cycle, minimizing any methodological impacts on the number of modules extracted (10). Lastly, it is important to note that the cutoffs identified by the classification tree algorithm are not intended to be used in a predictive model and applied generally. A much larger sample size with more balanced subgroups of individuals with two, and more than two modules would be required to establish generalizability of the cutoffs to other datasets.

In conclusion, our findings suggest that both CST and CRP are involved with locomotor recovery and the production of modules during steady state walking. Previous literature on upper extremity motor recovery has demonstrated greater expression of flexor or extensor synergies associated with involvement of CRP on the lesioned (72) as well as non-lesioned hemisphere (67, 68, 73), and this increased reliance on CRP structures may be associated with the amount of CST damage (74). However, there is limited understanding of a clear relationship between the extent of CST damage and ipsilesional or contralesional CRP involvement in independent control of modules during walking. In the current study we suggest that individuals with severe damage to CST and CRP on the lesioned hemisphere are likely to rely on the contralesional CRP and have two modules with severely impaired walking ability. Also, there is an indication of CST involvement in fractionation of modules associated with moderately impaired walking ability. Our results are consistent with bilateral CRP control leading to the two-module pattern in individuals with an intact CRP but damaged CST on the lesioned hemisphere. However, future studies are needed to determine the relationship between the role of bilateral CRP control and mass flexion extension co-excitation patterns. Therefore, influence of motor pathways from the lesioned and non-lesioned hemisphere on fractionation of modules and walking ability varies with the motor pathways affected by the lesion and the extent of damage or altered compensatory connectivity in these pathways.

## Data availability statement

The raw data supporting the conclusions of this article will be made available by the authors, without undue reservation.

## Ethics statement

The studies involving human participants were reviewed and approved by Institutional Review Board at Medical University of South Carolina. The patients/participants provided their written informed consent to participate in this study.

## Author contributions

SS, SK, TB, and RN have developed the concept and design of the study. SS, BS, MB, CG, NS, and CH have been involved with acquisition of data. SS, BS, BM, and LB have analyzed the data. SS, SK, JW, and LB have interpreted the results. SS, BS, and BM have drafted the manuscript. All co-authors have revised the manuscript critically to improve intellectual content and approved the final manuscript.

## Funding

This work was supported by the Rehabilitation Research & Development Service of the Department of Veterans Affairs through Grant 1I01RX001935 and Senior Research Career Scientist award through Grant 1IK6RX003075. This work was also supported in part by the NIH through P20-GM109040 and Promotion of Doctoral Studies I Scholarship from the Foundation for Physical Therapy Research.

## Conflict of interest

The authors declare that the research was conducted in the absence of any commercial or financial relationships that could be construed as a potential conflict of interest.

## Publisher's note

All claims expressed in this article are solely those of the authors and do not necessarily represent those of their affiliated organizations, or those of the publisher, the editors and the reviewers. Any product that may be evaluated in this article, or claim that may be made by its manufacturer, is not guaranteed or endorsed by the publisher.

## Author disclaimer

Any opinions expressed in this work are those of the authors and do not necessarily reflect the view of the U.S. Department of Veteran Affairs, or the NIH.

## References

[1] Twitchell TE. The restoration of motor function following hemiplegia in man. Brain. (1951) 74:443–80. 10.1093/brain/74.4.443 14895765

[2] Brunnstrom S. Motor testing procedures in hemiplegia: based on sequential recovery stages. Phys Ther. (1966) 46:357–75. 10.1093/ptj/46.4.357 5907254

[3] Clark DJ Ting LH Zajac FE Neptune RR Kautz SA. Merging of healthy motor modules predicts reduced locomotor performance and muscle coordination complexity post-stroke. J Neurophysiol. (2010) 103:844–57. 10.1152/jn.00825.2009 20007501 PMC2822696

[4] Fugl-Meyer AR Jääskö L Leyman I Olsson S Steglind S. The post-stroke hemiplegic patient. 1 a method for evaluation of physical performance. Scand J Rehabil Med. (1975) 7:13–31. 1135616

[5] Bowden MG Clark DJ Kautz SA. Evaluation of abnormal synergy patterns poststroke: relationship of the Fugl-Meyer Assessment to hemiparetic locomotion. Neurorehabil Neural Repair. (2010) 24:328–37. 10.1177/1545968309343215 19794132 PMC4434590

[6] Nadeau S Arsenault AB Gravel D Bourbonnais D. Analysis of the clinical factors determining natural and maximal gait speeds in adults with A Stroke1. Am J Phys Med Rehabil. (1999) 78:123–30. 10.1097/00002060-199903000-00007 10088586

[7] Rech KD Salazar AP Marchese RR Schifino G Cimolin V Pagnussat AS. Fugl-Meyer assessment scores are related with kinematic measures in people with chronic hemiparesis after stroke. J Stroke Cerebrovas Dis. (2020) 29:104463. 10.1016/j.jstrokecerebrovasdis.2019.104463 31740027

[8] McGowan CP Neptune RR Clark DJ Kautz SA. Modular control of human walking: adaptations to altered mechanical demands. J Biomech. (2010) 43:412–9. 10.1016/j.jbiomech.2009.10.009 19879583 PMC2813323

[9] Neptune RR Clark DJ Kautz SA. Modular control of human walking: a simulation study. J Biomech. (2009) 42:1282–7. 10.1016/j.jbiomech.2009.03.009 19394023 PMC2696580

[10] Seamon BA Neptune RR Kautz SA. Using a module-based analysis framework for investigating muscle coordination during walking in individuals poststroke: a literature review and synthesis. Appl Bionics Biomech. (2018) 2018:3795754. 10.1155/2018/3795754 29967653 PMC6008620

[11] Allen JL Kautz SA Neptune RR. The influence of merged muscle excitation modules on post-stroke hemiparetic walking performance. Clin Biomech. (2013) 28:697–704. 10.1016/j.clinbiomech.2013.06.003 23830138 PMC3732538

[12] Drew T. Motor cortical activity during voluntary gait modifications in the cat. I Cells related to the forelimbs. J Neurophysiol. (1993) 70:179–99. 10.1152/jn.1993.70.1.179 8360715

[13] Roh J Cheung VC Bizzi E. Modules in the brain stem and spinal cord underlying motor behaviors. J Neurophysiol. (2011). 10.1152/jn.00842.2010 21653716 PMC3174810

[14] Overduin SA. d'Avella A Carmena JM Bizzi E. Microstimulation activates a handful of muscle synergies. Neuron. (2012) 76:1071–7. 10.1016/j.neuron.2012.10.018 23259944 PMC3547640

[15] Capaday C Lavoie BA Barbeau H Schneider C Bonnard M. Studies on the corticospinal control of human walking. I. Responses to focal transcranial magnetic stimulation of the motor cortex. J Neurophysiol. (1999) 81:129–39. 10.1152/jn.1999.81.1.129 9914274

[16] Petersen TH Willerslev-Olsen M Conway BA Nielsen JB. The motor cortex drives the muscles during walking in human subjects. J Physiol. (2012) 590:2443–52. 10.1113/jphysiol.2012.227397 22393252 PMC3424763

[17] Schubert M Curt A Jensen L Dietz V. Corticospinal input in human gait: modulation of magnetically evoked motor responses. Exp Brain Res. (1997) 115:234–46. 10.1007/PL00005693 9224852

[18] Yakovenko S Krouchev N Drew T. Sequential activation of motor cortical neurons contributes to intralimb coordination during reaching in the cat by modulating muscle synergies. J Neurophysiol. (2011) 105:388–409. 10.1152/jn.00469.2010 21068260

[19] Drew T Kalaska J Krouchev N. Muscle synergies during locomotion in the cat: a model for motor cortex control. J Physiol. (2008) 586:1239–45. 10.1113/jphysiol.2007.146605 18202098 PMC2375657

[20] Ahn YH Ahn SH Kim H Hong JH Jang SH. Can stroke patients walk after complete lateral corticospinal tract injury of the affected hemisphere? Neuroreport. (2006) 17:987–90. 10.1097/01.wnr.0000220128.01597.e0 16791089

[21] Jang SH You SH Kwon Y-H Hallett M Lee MY Ahn MY. Cortical reorganization associated lower extremity motor recovery as evidenced by functional MRI and diffusion tensor tractography in a stroke patient. Restor Neurol Neurosci. (2005) 23:325–9. 16477094

[22] Jang SH Chang CH Lee J Kim CS Seo JP Yeo SS. Functional role of the corticoreticular pathway in chronic stroke patients. Stroke. (2013) 44:1099–104. 10.1161/STROKEAHA.111.000269 23444306

[23] York D. Review of descending motor pathways involved with transcranial stimulation. Neurosurgery. (1987) 20:70–3. 10.1097/00006123-198701000-00021 3543726

[24] Drew T Prentice S Schepens B. Cortical and brainstem control of locomotion. Prog Brain Res. (2004) 143:251–61. 10.1016/S0079-6123(03)43025-2 14653170

[25] Kautz SA Patten C Neptune RR. Does unilateral pedaling activate a rhythmic locomotor pattern in the nonpedaling leg in post-stroke hemiparesis? J Neurophysiol. (2006) 95:3154–63. 10.1152/jn.00951.2005 16452259

[26] Matsuyama K Mori F Nakajima K Drew T Aoki M Mori S. Locomotor role of the corticoreticular–reticulospinal–spinal interneuronal system. Prog Brain Res. (2004) 143:239–49. 10.1016/S0079-6123(03)43024-0 14653169

[27] Jang SH. The recovery of walking in stroke patients: a review. Int J Rehabil Res. (2010) 33:285–9. 10.1097/MRR.0b013e32833f0500 20805757

[28] Jang SH Lee SJ. Corticoreticular tract in the human brain: a mini review. Front Neurol. (2019) 10:1188. 10.3389/fneur.2019.01188 31803130 PMC6868423

[29] Drew T. Functional organization within the medullary reticular formation of the intact unanesthetized cat. III. Microstimulation during locomotion. J Neurophysiol. (1991) 66:919–38. 10.1152/jn.1991.66.3.919 1753295

[30] Kably B Drew T. Corticoreticular pathways in the cat. II. Discharge activity of neurons in area 4 during voluntary gait modifications. J Neurophysiol. (1998) 80:406–24. 10.1152/jn.1998.80.1.406 9658060

[31] Takakusaki K Chiba R Nozu T Okumura T. Brainstem control of locomotion and muscle tone with special reference to the role of the mesopontine tegmentum and medullary reticulospinal systems. J Neural Transm. (2016) 123:695–729. 10.1007/s00702-015-1475-4 26497023 PMC4919383

[32] Goldberg EJ Kautz SA Neptune RR. Can treadmill walking be used to assess propulsion generation? J Biomech. (2008) 41:1805–8. 10.1016/j.jbiomech.2008.03.009 18436229 PMC2413053

[33] Kautz SA Bowden MG Clark DJ Neptune RR. Comparison of motor control deficits during treadmill and overground walking poststroke. Neurorehabil Neural Repair. (2011) 25:756–65. 10.1177/1545968311407515 21636831 PMC4434587

[34] Bowden MG Balasubramanian CK Neptune RR Kautz SA. Anterior-posterior ground reaction forces as a measure of paretic leg contribution in hemiparetic walking. Stroke. (2006) 37:872–6. 10.1161/01.STR.0000204063.75779.8d 16456121

[35] Balasubramanian CK Bowden MG Neptune RR Kautz SA. Relationship between step length asymmetry and walking performance in subjects with chronic hemiparesis. Arch Phys Med Rehabil. (2007) 88:43–9. 10.1016/j.apmr.2006.10.004 17207674

[36] Burden A Bartlett R. Normalisation of EMG amplitude: an evaluation and comparison of old and new methods. Med Eng Phys. (1999) 21:247–57. 10.1016/S1350-4533(99)00054-5 10514043

[37] Halaki M Ginn K. Normalization of EMG signals: to normalize or not to normalize and what to normalize to. In:Naik GR, editor. Computational Intelligence in Electromyography Analysis-a Perspective on Current Applications and Future Challenges, Vol. 10. (2012), p. 49957. 10.5772/49957

[38] Rorden C Bonilha L Fridriksson J Bender B Karnath H-O. Age-specific CT and MRI templates for spatial normalization. Neuroimage. (2012) 61:957–65. 10.1016/j.neuroimage.2012.03.020 22440645 PMC3376197

[39] Ashburner J Friston KJ. Unified segmentation. Neuroimage. (2005) 26:839–51. 10.1016/j.neuroimage.2005.02.018 15955494

[40] Nachev P Coulthard E Jäger HR Kennard C Husain M. Enantiomorphic normalization of focally lesioned brains. Neuroimage. (2008) 39:1215–26. 10.1016/j.neuroimage.2007.10.002 18023365 PMC2658465

[41] Yeo SS Chang MC Kwon YH Jung YJ Jang SH. Corticoreticular pathway in the human brain: diffusion tensor tractography study. Neurosci Lett. (2012) 508:9–12. 10.1016/j.neulet.2011.11.030 22197953

[42] Yeo SS Jang SH Park GY Oh S. Effects of injuries to descending motor pathways on restoration of gait in patients with pontine hemorrhage. J Stroke Cerebrovasc Dis. (2020) 29:104857. 10.1016/j.jstrokecerebrovasdis.2020.104857 32409256

[43] Soulard J Huber C Baillieul S Thuriot A Renard F Broche BA. Motor tract integrity predicts walking recovery: a diffusion MRI study in subacute stroke. Neurology. (2020) 94:e583–93. 10.1212/WNL.0000000000008755 31896618

[44] Bonilha L Nesland T Rorden C Fillmore P Ratnayake RP Fridriksson J. Mapping remote subcortical ramifications of injury after ischemic strokes. Behav Neurol. (2014) 2014:215380. 10.1155/2014/215380 24868120 PMC4017848

[45] Bonilha L Nesland T Rorden C Fridriksson J. Asymmetry of the structural brain connectome in healthy older adults. Front Psychiatry. (2014) 4:186. 10.3389/fpsyt.2013.00186 24409158 PMC3885898

[46] Andersson JLR Sotiropoulos SN. An integrated approach to correction for off-resonance effects and subject movement in diffusion MR imaging. Neuroimage. (2016) 125:1063–78. 10.1016/j.neuroimage.2015.10.019 26481672 PMC4692656

[47] Behrens TE Berg HJ Jbabdi S Rushworth MF Woolrich MW. Probabilistic diffusion tractography with multiple fibre orientations: what can we gain? Neuroimage. (2007) 34:144–55. 10.1016/j.neuroimage.2006.09.018 17070705 PMC7116582

[48] Hernandez M Guerrero GD Cecilia JM Garcia JM Inuggi A Jbabdi S. Accelerating fibre orientation estimation from diffusion weighted magnetic resonance imaging using GPUs. PLoS ONE. (2013) 8:e61892. 10.1371/journal.pone.0061892 23658616 PMC3643787

[49] Wilmskoetter J Bonilha L Martin-Harris B Elm JJ Horn J Bonilha HS. Mapping acute lesion locations to physiological swallow impairments after stroke. Neuroimage Clin. (2019) 22:101685. 10.1016/j.nicl.2019.101685 30711683 PMC6357850

[50] Kim NY Lee SC Shin J-C Park JE Kim YW. Voxel-based lesion symptom mapping analysis of depressive mood in patients with isolated cerebellar stroke: a pilot study. Neuroimage Clin. (2017) 13:39–45. 10.1016/j.nicl.2016.11.011 27942446 PMC5133641

[51] Achilles EI Weiss PH Fink GR Binder E Price CJ Hope TM. Using multi-level Bayesian lesion-symptom mapping to probe the body-part-specificity of gesture imitation skills. Neuroimage. (2017) 161:94–103. 10.1016/j.neuroimage.2017.08.036 28822751 PMC5692920

[52] Sperber C Nolingberg C Karnath HO. Post-Stroke Cognitive Deficits Rarely Come Alone: Handling Co-morbidity in Lesion-Behaviour Mapping. Hoboken, NJ: Wiley Online Library (2020). Report No.: 1065-9471. 10.1002/hbm.24885 PMC7267998 31782852

[53] Johnson WE Li C Rabinovic A. Adjusting batch effects in microarray expression data using empirical Bayes methods. Biostatistics. (2007) 8:118–27. 10.1093/biostatistics/kxj037 16632515

[54] Fortin J-P Parker D Tunç B Watanabe T Elliott MA Ruparel K. Harmonization of multi-site diffusion tensor imaging data. Neuroimage. (2017) 161:149–70. 10.1016/j.neuroimage.2017.08.047 28826946 PMC5736019

[55] Routson RL Clark DJ Bowden MG Kautz SA Neptune RR. The influence of locomotor rehabilitation on module quality and post-stroke hemiparetic walking performance. Gait Posture. (2013) 38:511–7. 10.1016/j.gaitpost.2013.01.020 23489952 PMC3687005

[56] Routson RL Kautz SA Neptune RR. Modular organization across changing task demands in healthy and poststroke gait. Physiol Rep. (2014) 2:e12055. 10.14814/phy2.12055 24963035 PMC4208640

[57] Duncan PW Propst M Nelson SG. Reliability of the Fugl-Meyer assessment of sensorimotor recovery following cerebrovascular accident. Phys Ther. (1983) 63:1606–10. 10.1093/ptj/63.10.1606 6622535

[58] Duncan PW Sullivan KJ Behrman AL Azen SP Wu SS Nadeau SE. Body-weight–supported treadmill rehabilitation after stroke. N Engl J Med. (2011) 364:2026–36. 10.1056/NEJMoa1010790 21612471 PMC3175688

[59] Dobkin BH Firestine A West M Saremi K Woods R. Ankle dorsiflexion as an fMRI paradigm to assay motor control for walking during rehabilitation. Neuroimage. (2004) 23:370–81. 10.1016/j.neuroimage.2004.06.008 15325385 PMC4164211

[60] Jayaram G Stagg CJ Esser P Kischka U Stinear J Johansen-Berg H. Relationships between functional and structural corticospinal tract integrity and walking post stroke. Clin Neurophysiol. (2012) 123:2422–8. 10.1016/j.clinph.2012.04.026 22717679 PMC3778984

[61] Jang SH Kim K Kim SH Son SM Jang WH Kwon HG. The relation between motor function of stroke patients and diffusion tensor imaging findings for the corticospinal tract. Neurosci Lett. (2014) 572:1–6. 10.1016/j.neulet.2014.04.044 24796808

[62] Lemon RN. Descending pathways in motor control. Annu Rev Neurosci. (2008) 31:195–218. 10.1146/annurev.neuro.31.060407.125547 18558853

[63] Zaaimi B Dean LR Baker SN. Different contributions of primary motor cortex, reticular formation, and spinal cord to fractionated muscle activation. J Neurophysiol. (2018) 119:235–50. 10.1152/jn.00672.2017 29046427 PMC5866475

[64] Smith V Maslovat D Drummond NM Hajj J Leguerrier A Carlsen AN. High-intensity transcranial magnetic stimulation reveals differential cortical contributions to prepared responses. J Neurophysiol. (2019) 121:1809–21. 10.1152/jn.00510.2018 30864866 PMC6589715

[65] Maslovat D Teku F Smith V Drummond NM Carlsen AN. Bimanual but not unimanual finger movements are triggered by a startling acoustic stimulus: evidence for increased reticulospinal drive for bimanual responses. J Neurophysiol. (2020) 124:1832–8. 10.1152/jn.00309.2020 33026906

[66] Xu J Ejaz N Hertler B Branscheidt M Widmer M Faria AV. Separable systems for recovery of finger strength and control after stroke. J Neurophysiol. (2017) 118:1151–63. 10.1152/jn.00123.2017 28566461 PMC5547267

[67] McPherson JG Chen A Ellis MD Yao J Heckman C Dewald JP. Progressive recruitment of contralesional cortico-reticulospinal pathways drives motor impairment post stroke. J Physiol. (2018) 596:1211–25. 10.1113/JP274968 29457651 PMC5878212

[68] Karbasforoushan H Cohen-Adad J Dewald JP. Brainstem and spinal cord MRI identifies altered sensorimotor pathways post-stroke. Nat Commun. (2019) 10:1–7. 10.1038/s41467-019-11244-3 31388003 PMC6684621

[69] Rouiller E Yu X Moret V Tempini A Wiesendanger M Liang F. Dexterity in adult monkeys following early lesion of the motor cortical hand area: the role of cortex adjacent to the lesion. Eur J Neurosci. (1998) 10:729–40. 10.1046/j.1460-9568.1998.00075.x 9749734

[70] Baker SN Zaaimi B Fisher KM Edgley SA Soteropoulos DS. Pathways mediating functional recovery. Prog Brain Res. (2015) 218:389–412. 10.1016/bs.pbr.2014.12.010 25890147

[71] Oliveira AS Gizzi L Farina D Kersting UG. Motor modules of human locomotion: influence of EMG averaging, concatenation, and number of step cycles. Front Hum Neurosci. (2014) 8:335. 10.3389/fnhum.2014.00335 24904375 PMC4033063

[72] Schulz R Park E Lee J Chang WH Lee A Kim Y-H. Synergistic but independent: the role of corticospinal and alternate motor fibers for residual motor output after stroke. Neuroimage Clin. (2017) 15:118–24. 10.1016/j.nicl.2017.04.016 28516034 PMC5426012

[73] Bradnam LV Stinear CM Byblow WD. Ipsilateral motor pathways after stroke: implications for non-invasive brain stimulation. Front Hum Neurosci. (2013) 7:184. 10.3389/fnhum.2013.00184 23658541 PMC3647244

[74] Choudhury S Shobhana A Singh R Sen D Anand SS Shubham S. The relationship between enhanced reticulospinal outflow and upper limb function in chronic stroke patients. Neurorehabil Neural Repair. (2019) 33:375–83. 10.1177/1545968319836233 30913964

